# P-glycoprotein is not expressed in a majority of colorectal carcinomas and is not regulated by mutant p53 in vivo.

**DOI:** 10.1038/bjc.1995.329

**Published:** 1995-08

**Authors:** P. De Angelis, T. Stokke, L. Smedshammer, R. A. Lothe, G. Lehne, Y. Chen, O. P. Clausen

**Affiliations:** Institute of Pathology, Norwegian National Hospital, Oslo.

## Abstract

**Images:**


British Journal of Cancer (1995) 72, 307-311
© 1995 Stockton Press All rights reserved 0007-0920/95 $12.00

### P-glycoprotein is not expressed in a majority of colorectal carcinomas and is not regulated by mutant p53 *in vivo*

P De Angelis[1], T Stokke[2], L Smedshammer[2], RA Lothe[3], G Lehne[4], Y Chen[1] and OPF Clausen[1]

[1]*Institute of Pathology The Norwegian National Hospital, Oslo, Norway; Departments of* [2]*Biophysics and* [3]*Genetics, Institute for Cancer Research, The Norwegian Radium Hospital, Oslo, Norway;* [4]*Institute of Clinical Pharmacology, The Norwegian National Hospital, Oslo, Norway.*

**Summary** Overexpression of the *MDR1* product, P-glycoprotein (Pgp), has been shown to be one of the mechanisms underlying the development of multidrug resistance (MDR). Recently, one mutant p53 has been shown to stimulate the *MDR1* gene promoter *in vitro*, whereas wild-type p53 repressed this activity. We measured Pgp and p53 expression by immunoblotting in 34 colorectal tumours, and performed mutation analyses on the p53-positive tumours to confirm the presence of mutant p53 protein. Tumour DNA indices (DIs) were also measured using flow cytometry. Pgp was detected in 44% (15/34) of the tumours and in 100% (13/13) of the normal mucosas ($P = 0.0005$), with highest levels of expression seen in normal mucosa, suggesting that initial drug resistance in colorectal tumours is not caused by Pgp. Highly DNA aneuploid tumours demonstrated the lowest levels of Pgp expression relative to moderately aneuploid and diploid colorectal tumours. p53 protein was detected in 53% (18/34) of the tumours, and 12 of 14 p53-positive tumours had p53 gene mutations. p53-negative tumours had approximately twice the level of Pgp expression of p53-positive tumours. Pgp expression was not associated with either p53 expression ($P = 0.73$) or incidence of p53 gene mutation ($P = 0.70$), suggesting that mutant p53 does not induce Pgp overexpression in colorectal carcinomas.

**Keywords:** P-glycoprotein; mutant p53; tumour DNA ploidy; colorectal tumours

A majority of colorectal carcinomas and other solid tumours are resistant to cytostatic drugs, which is a significant problem in their treatment (de Vries and Pinedo, 1991). The cause of such resistance may be determined not only by specific cellular mechanisms such as the multidrug resistance (MDR) phenotype associated with overexpression of the *MDR1* gene, but also by characteristics of the tumour population, such as the proportion of quiescent cells and adequacy of blood supply (Judson, 1992).

The *MDR1* gene product, a 170 kDa protein known as P-glycoprotein (Pgp), appears to be a bifunctional transmembrane protein that functions both as an energy-dependent drug efflux pump of broad specificity (e.g. for anthracyclines and other natural hydrophobic compounds) and as a chloride channel (Gill *et al.*, 1992). Several studies have shown that MDR can be conferred upon drug-sensitive cells by transfer of genes encoding Pgp (Gros *et al.*, 1986; Ueda *et al.*, 1987). *MDR1* RNA and Pgp are found at substantial levels in normal colon, small intestine, kidney, liver and adrenal gland (Fojo *et al.*, 1987), suggesting a normal transporter role for Pgp in these tissues. Pgp has also been found in untreated human cancers derived from normal tissues that express Pgp, such as carcinomas of the colon, liver, kidney, pancreas and adrenal gland (Goldstein *et al.*, 1989). However, it has also been shown that expression of low constitutive levels of *MDR1* mRNA/Pgp may not necessarily result in the functional expression of the MDR phenotype (Hamada *et al.*, 1987; Chambers *et al.*, 1990; Kramer *et al.*, 1993).

Chin *et al.* (1992) have recently shown that one mutant human p53 protein has a specific stimulatory effect upon the *MDR1* gene promoter in 3T3 cells and that wild-type p53 exerts specific repression. Expression of p53 protein in colorectal carcinomas is associated with a high degree of tumour DNA aneuploidy (Carder *et al.*, 1993; De Angelis *et al.*, 1993) and a high incidence of p53 gene mutation (De Angelis *et al.*, 1993). It could be speculated that highly aneuploid tumours with mutant p53 express excess Pgp as a result of the stimulatory effect of p53 on the *MDR1* gene. In this study we have measured Pgp expression and p53 expression in 34 colorectal tumours in order to determine whether Pgp expression is likely to confer initial drug resistance and whether mutant p53 stimulates Pgp expression *in vivo*.

### Materials and methods

### *Clinical material*

Thirty-four surgically removed and previously untreated colorectal carcinomas and 13 normal colonic mucosas were collected and studied. All tumours were classified according to Dukes' stage (Dukes, 1932) and histological grade (Jass *et al.*, 1986) (Table I, includes tumour site). Normal mucosa samples were selected from macroscopically normal areas of surgical specimens. Fresh tumour (T) and normal mucosa (N) samples were frozen without buffer at $-80°C$ immediately after surgery. During tissue sectioning and preparation procedures for DNA ploidy analysis and immunoblotting, the tumours and normal mucosas were kept on dry ice until used.

DNA indices (DIs) were determined for the tumour set using laser flow cytometry as described previously (De Angelis *et al.*, 1993), and are presented in Table I. The DI characterises a tumour as either DNA aneuploid ($DI > 1.00$) or diploid ($DI = 1.00$) (Hiddemann *et al.*, 1984; Dressler and Bartow, 1989). Highly DNA aneuploid tumours were defined as those with a $DI \geqslant 1.30$, and moderately DNA aneuploid (hyperdiploid) tumours as those with a $DI > 1.00$ and $< 1.30$. The DI has been used as a prognostic parameter (with modest impact, Bauer *et al.*, 1993) in addition to Dukes' stage and histological grade classifications for the prediction of prognosis of patients with colorectal tumours.

### *Immunoblotting*

Slices (approximately 0.5–1.0 mm thick) of tumour and normal mucosa were cut with scalpels and boiled for 3–4 min in sodium dodecyl sulphate (SDS) sample buffer (Laemmli,

Correspondence: P De Angelis, The Norwegian National Hospital, Institute for Pathology, Pilestredet 32, 0027 Oslo, Norway
Received 9 December 1994; revised 27 March 1995; accepted 3 April 1995

**Table I** Tumour site, Dukes' stage, histological grade and DNA index (DI) for 34 colorectal tumours

| *Tumour* | *Tumour site* | *Dukes' stage* | *Histological grade* | *DNA index (DI)* |
|---|---|---|---|---|
| 90-1 | Left colon | D | PD | 1.21 |
| 90-4 | Right colon | C | MD | 1.00 |
| 90-7 | Right colon | D | PD | 1.10 |
| 90-8 | Rectum | C | MD | 1.00 |
| 90-9 | Left colon | B | MD | 1.22 |
| 90-10 | Right colon | B | MD | 1.00 |
| 90-19 | Rectum | B | MD | 1.77 |
| 90-20 | Right colon | B | MD | 1.26 |
| 92-1 | Left colon | C | MD | 1.70 |
| 92-2 | Left colon | D | MD | 1.37 |
| 92-4 | Right colon | B | MD | 1.00 |
| 92-5 | Left colon | B | MD | 1.49 |
| 92-6 | Right colon | D | PD | 1.31 |
| 92-8 | Left colon | D | HD | 1.60 |
| 92-9 | Right colon | B | MD | 1.15 |
| 92-10 | Right colon | B | MD | 1.00 |
| 94-1 | Left colon | B | MD | 1.56 |
| 94-3 | Rectum | B | MD | 1.00 |
| 94-6 | Left colon | D | PD | 1.71 |
| 94-7 | Rectum | A | MD | 1.76 |
| 94-8 | Left colon | C | MD | 1.94 |
| 94-9 | Left colon | B | MD | 1.78 |
| 94-10 | Rectum | D | MD | 1.97 |
| 94-13 | Rectum | B | MD | 1.59 |
| 94-14 | Rectum | B | MD | 1.00 |
| 94-15 | Rectum | C | MD | 1.58 |
| 94-16 | Left colon | C | PD | 1.39 |
| 94-17 | Rectum | B | MD | 1.00 |
| 94-18 | Left colon | C | MD | 1.69 |
| 94-19 | Right colon | D | PD | 1.00 |
| 94-21 | Right colon | D | MD | 1.74 |
| 94-23 | Left colon | B | MD | 1.20 |
| 94-24 | Rectum | D | MD | 1.70 |
| 94-25 | Rectum | B | MD | 1.00 |

PD, poorly differentiated; MD, moderately differentiated; HD, highly differentiated.

1970) containing protease inhibitors. Representative slices were also cut from the same areas of the tumours as used for immunoblotting, processed for routine histology, and examined by one of us (OPFC) in order to estimate the percentages of tumour and normal mucosal cells in each section. The mean percentage of tumour cells for all sections examined ($n = 31$) was 90.4% ± 12.6%.

The proteins were first separated by electrophoresis in 8.0% SDS-polyacrylamide gels (Laemmli, 1970). Immunoblotting was done as described previously (De Angelis *et al.*, 1993) using the anti-Pgp monoclonal antibody C219 (Centocor, PA, USA), the anti-p53 monoclonal antibodies PAb 1801 and PAb 421, and the anti-p83 monoclonal antibody 34C1 (courtesy of T Stokke). The amount of p83 (nucleoplasmic protein expressed at equivalent levels in proliferating and non-proliferating cells; T Stokke and S Funderud, unpublished) was determined on the same blots in order to control for gel loading, cell concentration and protein degradation. A biotin–streptavidin alkaline phosphatase staining procedure was used to detect the primary antibodies (Amersham, UK). The amounts of the different proteins were evaluated by densitometry using ImageQuant densitometry software (Molecular Dynamics, USA) to analyse stored gel images generated with an Agfa photo scanner (Agfa, Germany). Levels of protein expression are reported relative to p83 expression.

### *Mutation analysis within the p53 gene*

DNA from 28 colorectal tumour samples was subjected to mutation analysis using polymerase chain reaction (PCR) followed by constant denaturant gel electrophoresis (CDGE). We screened for mutations in the conserved domains corresponding to the following codons: codons 128–153 and 155–185 of exon 5, codons 237–253 of exon 7 and codons 265–301 of exon 8. Twenty tumours were also analysed for mutations in codon region 189–215 of exon 6. The optimal conditions for PCR and CDGE have been described elsewhere (Borresen *et al.*, 1991; Smith-Sorensen *et al.*, 1993).

### *DNA sequencing*

Tumour samples shown to be mutated by CDGE were submitted to direct sequencing in order to determine exactly which codon was affected. A new PCR product was made using primers outside the ones used for CDGE. The PCR products were sequenced directly with standard dideoxy-sequencing reactions using Dynabeads M280–streptavidin (Dynal AS, Oslo, Norway) as solid support (Hultman *et al.*, 1989). It has previously been shown that CDGE is a more sensitive method of detecting mutants than this type of sequencing (Borresen *et al.*, 1991). Therefore not all mutants found by CDGE were confirmed by sequencing.

### *Statistical analysis*

Fisher's two-tailed 2 × 2 contingency test was used to check for significance of correlations (*P*-values) between any two parameters. *P*-values $<0.05$ were considered to denote a statistically significant difference.

### Results

Pgp was detected in 44% (15/34) of the tumours and in 100% (13/13) of the normal mucosas examined (Figure 1; Table II) ($P = 0.0005$), with higher levels of expression seen in normal mucosa generally (mean expression 0.260 ± 0.22) as assessed by densitometry, in contrast to a mean expression of 0.061 ± 0.12 for all of the tumours. Table III demonstrates the lower levels of Pgp expression in a subset of the tumour group compared with the levels seen in the corresponding normal mucosas; it was observed in two cases (92-5 and 92-9)

**Figure 1** Immunoblot of colorectal tumours 92-1, 92-2, 92-4 to 92-6, and corresponding normal mucosas 92-1N, 92-2N, 92-4N to 92-6N, stained for Pgp, p83 and p53. The blot to the left is the control blot (without primary antibodies) for non-specific staining. The amounts of these proteins were quantified by densitometry and the results presented as fractional values relative to p83 (Tables II and III). Normal mucosa 92-5N expressed low levels of Pgp as demonstrated by the weakly stained Pgp band. The strong p83 bands for both the tumour and normal mucosal samples indicate lack of protein degradation and that relatively equal amounts of protein were loaded per lane.

that the level of Pgp expression in the tumours was higher than in the corresponding normal mucosas.

There was significant association neither between Pgp expression and Dukes' stage (A plus B *vs* C plus D; $P = 0.51$) nor between Pgp expression and tumour site in the colon ($P = 1.00$, right colon *vs* left colon/rectum). Additionally, there was not a significant association between Pgp expression and histological grade ($P = 1.00$, poorly differentiated *vs* moderately and highly differentiated tumours).

Fifty per cent (12/24) of the aneuploid tumours were positive for Pgp, compared with 40% (4/10) of the diploid tumours ($P = 0.71$). The densitometry results demonstrated a trend which showed that the highly aneuploid tumours expressed the lowest amounts of Pgp (mean = 0.034 ± 0.08), the moderately aneuploid tumours more than double that seen in the highly aneuploid tumours (mean = 0.086 ± 0.09), the diploid tumours approximately the same amounts as those seen in the moderately aneuploid tumours (mean = 0.078 ± 0.19) and the normal mucosas the highest amounts of Pgp (mean = 0.260 ± 0.22). However, this trend was not significant since the standard deviations for each sample group were high and overlapped with the other groups, and because each of the sample groups tended to be small in size.

p53 was detected in 53% (18/34) of the tumours (Figure 1; Table II), whereas none of the normal mucosas expressed p53. Twelve of 14 p53-positive tumours examined for mutations had p53 gene mutations.

Pgp expression was significantly associated neither with p53 expression ($P = 0.73$) nor with incidence of p53 gene mutations ($P = 0.70$). The level of Pgp expression in the p53-positive tumours was determined to be 0.043 ± 0.087, approximately half the level of expression seen for the p53-negative tumours (0.081 ± 0.156, although this difference was not significant because of the high and overlapping standard deviations for each group.

### Discussion

Pgp was expressed in 44% of the colorectal tumours and in all of the normal mucosas examined in this study, with the highest levels of expression seen in normal mucosa. The positive tumours generally expressed less Pgp than normal mucosa. Our results are in agreement with those of a previous study (Fujii *et al.*, 1993), which demonstrated that 44% of colon carcinomas were positive for Pgp by immunofluorescence analyses using both flow cytometry and immunohistochemistry in sections, as well as with previous studies demonstrating Pgp expression generally in normal colonic mucosa (Fojo *et al.*, 1987) and colonic tumours (Goldstein *et al.*, 1989). Peters *et al.* (1992) found positive Pgp expression in all but one tumour; the tumours had significantly higher levels of Pgp than the normal mucosas.

**Table III** Pgp is not expressed in many tumours relative to the corresponding normal mucosas

| *Tumour* | *Fraction Pgp expressed in normal mucosa* | *Fraction Pgp expressed in tumour* |
|---|---|---|
| 90-1 | 0.576 | 0.124 |
| 90-7 | 0.074 | 0.0 |
| 90-8 | 0.059 | 0.037 |
| 90-10 | 0.733 | 0.0 |
| 92-1 | 0.269 | 0.0 |
| 92-2 | 0.207 | 0.0 |
| 92-4 | 0.298 | 0.128 |
| 92-5 | 0.091 | 0.324 |
| 92-6 | 0.344 | 0.0 |
| 92-8 | 0.131 | 0.112 |
| 92-9 | 0.076 | 0.211 |
| Mean ± s.d. | 0.260 ± 0.22 | 0.085 ± 0.11 |

**Table II** Pgp and p53 status for 34 colorectal tumours

| *Tumour* | *Fraction Pgp expressed* | *Pgp status* | *Fraction p53 expressed* | *p53 status* | *p53 Gene mutations* Exon codon | Mutation | *Amino acid* |
|---|---|---|---|---|---|---|---|
| 90-1 | 0.124 | + | 0.0 | – | | | |
| 90-4 | 0.610 | ++ | 0.0 | – | | | |
| 90-7 | 0.0 | – | 0.0 | – | No mutation revealed | | |
| 90-8 | 0.037 | + | 0.0 | – | No mutation revealed | | |
| 90-9 | 0.013 | + | 0.0 | – | No mutation revealed | | |
| 90-10 | 0.0 | – | 0.0 | – | No mutation revealed | | |
| 90-19 | 0.191 | + | 0.171 | + | | | |
| 90-20 | 0.025 | + | 0.0 | – | No mutation revealed | | |
| 92-1 | 0.0 | – | 0.232 | + | Exon 5: codon 175 | CGC to CAC | Arg to His |
| 92-2 | 0.0 | – | 0.724 | ++ | Exon 8: codon 275 | TGT to TTT | Cys to Phe |
| 92-4 | 0.128 | + | 0.0 | – | No mutation revealed | | |
| 92-5 | 0.324 | ++ | 0.860 | ++ | Exon 8: unspecified codon | | |
| 92-6 | 0.0 | – | 0.457 | ++ | Exon 8: codon 282 | CGG to TGG | Arg to Trp |
| 92-8 | 0.112 | + | 0.910 | ++ | Exon 8: codon 273 | CGT to CAT | Arg to His |
| 92-9 | 0.211 | ++ | 0.0 | – | No mutation revealed | | |
| 92-10 | 0.0 | – | 0.029 | – | | | |
| 94-1 | 0.0 | – | 0.0 | – | No mutation revealed | | |
| 94-3 | 0.0 | – | 0.0 | – | No mutation revealed | | |
| 94-6 | 0.012 | + | 0.162 | + | Exon 5: unspecified codon | | |
| 94-7 | 0.0 | – | 0.206 | + | | | |
| 94-8 | 0.056 | + | 0.127 | + | Exon 7: codon 245 | GGC to AGC | Gly to Ser |
| 94-9 | 0.015 | + | 0.216 | + | Exon 5: unspecified codon | | |
| 94-10 | 0.062 | + | 0.240 | + | | | |
| 94-13 | 0.0 | – | 0.270 | + | Exon 5: codon 130 | CTC to CAC | Leu to His |
| 94-14 | 0.0 | – | 0.113 | + | Exon 5: codon 176 | TGC to TAC | Cys to Tyr |
| 94-15 | 0.0 | – | 0.0 | – | Exon 6: codon 196 | CGA to TGA | Arg to STOP |
| 94-16 | 0.0 | – | 0.042 | + | | | |
| 94-17 | 0.0 | – | 0.062 | + | No mutation revealed | | |
| 94-18 | 0.0 | – | 0.116 | + | Exon 5: unspecified codon | | |
| 94-19 | 0.0 | – | 0.195 | + | No mutation revealed | | |
| 94-21 | 0.0 | – | 0.269 | ++ | Exon 6: unspecified codon | | |
| 94-23 | 0.142 | ++ | 0.0 | – | No mutation revealed | | |
| 94-24 | 0.0 | – | 0.0 | – | Exon 5: unspecified codon | | |
| 94-25 | 0.0 | – | 0.0 | – | No mutation revealed | | |

In the columns 'Pgp status' and 'p53 status', the amounts of each protein were visually evaluated from the immunoblots and described as absent (–), present in low amounts (+) and present in high amounts (++).

This discrepancy could be explained by their lack of gel loading/concentration standards when quantifying Pgp expression (p83 in our study) and different methods of isolation of cells from tumour and mucosal samples.

More than half of the colorectal tumours examined in this study were negative for Pgp expression, possibly reflecting their clonal origin from a cell which did not originally express Pgp (Noonan *et al.*, 1990). This Pgp-negative cell might be a crypt epithelial cell, since these have been shown to be negative for Pgp expression, whereas the surface epithelial cells lining the lumen (apical brush border cells) are Pgp-positive (Cordon-Cardo *et al.*, 1990). This reflects a differentiation-dependent pattern of expression as normal mucosal cells move up the crypt toward the surface. We estimated the percentage of normal mucosal cells in the tumour sections which might influence the number of Pgp-positive tumours scored. We are confident that the immunoblotting results reflect the biological characteristics of tumour cells, since very few non-tumour epithelial cells were seen in the sections examined (<10%) from the same areas of the tumours used for immunoblotting. Several tumours in our study which contained some normal cells were not scored as positive, thus contamination is not a significant problem in this study. It may mean, however, that heterogeneous tumours with a low number of Pgp-positive cells may be scored as Pgp-negative. Heterogeneity of Pgp staining and tumour sampling are other factors which may influence the number of positive tumours scored. However, these factors do not influence our main conclusion, which is that colorectal tumours either do not express Pgp at all (lack of differentiated phenotype) or do so to a much lesser extent than normal mucosa.

There was no correlation of Pgp expression with Dukes' stage, histological grade, or tumour site, confirming the results of Pirker *et al.* (1993). We also found no significant correlation between Pgp expression and DNA aneuploidy, consistent with the results of Sinicrope *et al.* (1994). However, these results are not in agreement with those of Danova *et al.* (1992), who demonstrated a correlation between DNA aneuploidy and higher levels of Pgp expression relative to diploid tumours and normal tissue.

Chin *et al.* (1992) found that one mutant human p53 protein (point mutation at codon 175, Arg to His substitution) had a specific stimulatory effect upon the *MDR1* gene promoter in 3T3 cells, suggesting that colon tumours which express mutant p53 should overexpress Pgp (relative to the levels of Pgp expressed in normal mucosa). However, we found no association between mutant p53 and Pgp expression. Where there was Pgp expression in the p53-positive tumours, it was still lower than that seen in normal mucosa. Additionally, tumour 92-1 in our tumour set expressed the particular codon 175 mutant p53 as studied by Chin *et al.* (1992), but did not express Pgp. This independence of Pgp and mutant p53 expression is supported by recent studies of B-cell chronic lymphocytic leukaemia (El Rouby *et al.*, 1993), acute myelogenous leukaemia (Zhao *et al.*, 1992) and myelodysplastic syndromes (Preudhomme *et al.*, 1993), which demonstrated that *MDR1* gene overexpression is independent of p53 gene mutations/mutant p53 protein.

It was not possible in this study directly to correlate Pgp expression in colon carcinomas with response to cytostatics and overall survival, since only one of the patients received chemotherapy for his tumour. However, the lack of Pgp expression in many colon tumours suggests that resistance mechanisms other than MDR are responsible for the initial resistance to cytostatic drugs often seen in colon tumours. Non-MDR mechanisms which have been shown to be involved in mediating resistance to cytostatic drugs (other than anthracyclines) in colon tumours are the activity of glutathione *S*-transferase (Waxman, 1990; Clapper *et al.*, 1991; De Waziers *et al.*, 1991; Moorghen *et al.*, 1991; Peters *et al.*, 1992) and the alteration of DNA-associated topoisomerase II which is involved in post-replication repair (Redmond *et al.*, 1991).

**Acknowledgements**
The authors wish to thank Erik Trondsen MD of the Surgical Department, Akershus Hospital, Oslo, Norway, for providing colorectal tumour and mucosal specimens, and Anne Cromarty for excellent technical assistance. This work was supported by the Norwegian Cancer Society and the Norwegian Research Council for Science and Humanities.

### References

BAUER KD, BAGWELL CB, GIARETTI W, MELAMED M, ZARBO RD, WITZIG TE AND RABINOVITCH P. (1993). Consensus review of the clinical utility of DNA flow cytometry in colorectal cancer. *Cytometry*, **14**, 486–491.

BORRESEN A-L, HOVIG E, SMITH-SORENSEN B, MALKIN D, LYSTAD S, ANDERSEN TI, NESLAND JM, ISSELBACHER KJ AND FRIEND SH. (1991). Constant denaturant gel electrophoresis as a rapid screening technique for p53 mutations. *Proc. Natl Acad. Sci. USA*, **88**, 8405–8409.

CARDER P, WYLLIE AH, PURDIE CA, MORRIS RG, WHITE S, PIRIS J AND BIRD CC. (1993). Stabilised p53 facilitates aneuploid clonal divergence in colorectal cancer. *Oncogene*, **8**, 1397–1401.

CHAMBERS TC, MCAVOY EM, JACOBS JW AND EILON G. (1990). Protein kinase C phosphorylates P-glycoprotein in multidrug resistant human KB carcinoma cells. *J. Biol. Chem.*, **265**, 7679–7686.

CHIN K-V, UEDA K, PASTAN I AND GOTTESMAN MM. (1992). Modulation of activity of the promoter of the human MDR1 gene by Ras and p53. *Science*, **255**, 459–462.

CLAPPER ML, HOFFMAN SJ AND TEW KD. (1991). Glutathione S-transferases in normal and malignant human colon tissue. *Biochim. Biophys. Acta*, **1096**, 209–216.

CORDON-CARDO C, O'BRIEN JP, BOCCIA J, CASALS D, BERTINO JR AND MELAMED MR. (1990). Expression of the multidrug resistance gene product (P-glycoprotein) in human normal and tumor tissues. *J. Histochem. Cytochem.*, **38**, 1277–1287.

DANOVA M, GIORDANO M, ERBA E, PALMERI S, CANDILORO V, RICCARDI A, UCCI G, MAZZINI G, D'INCALCI MD AND ASCARI E. (1992). Flow cytometric analysis of multidrug-resistance-associated antigen (P-glycoprotein) and DNA ploidy in human colon cancer. *J. Cancer Res. Clin. Oncol.*, **118**, 575–580.

DE ANGELIS P, STOKKE T, SMEDSHAMMER L, LOTHE RA, MELING GI, ROFSTAD M, CHEN Y AND CLAUSEN OPF. (1993). p53 expression is associated with a high degree of tumor DNA aneuploidy and incidence of p53 mutation, and is localized to the aneuploid component in colorectal carcinomas. *Int. J. Oncol.*, **3**, 305–312.

DE VRIES EG AND PINEDO HM. (1991). Clinical implications of multidrug resistance to chemotherapy. *Cancer Treat. Res.*, **57**, 171–186.

DE WAZIERS I, CUGNENC PH, BERGER A, LEROUX JP AND BEAUNE PH. (1991). Drug-metabolizing enzyme expression in human normal, peri-tumoral and tumoral colorectal tissue samples. *Carcinogenesis*, **12**, 905–909.

DRESSLER L AND BARTOW SA. (1989). DNA flow cytometry in solid tumors: practical aspects and clinical applications. *Semin. Diag. Pathol.*, **6**, 55–82.

DUKES CE. (1932). The classification of cancer of the rectum. *J. Pathol. Bacteriol.*, **35**, 323–332.

EL ROUBY S, THOMAS A, COSTIN D, ROSENBERG CR, POTMESIL M, SILBER R AND NEWCOMB EW. (1993). p53 gene mutation in B-cell chronic lymphocytic leukemia is associated with drug resistance and is independent of MDR1/MDR3 gene expression. *Blood*, **82**, 3452–3459.

FOJO AT, UEDA K, SLAMON DJ, POPLACK DG, GOTTESMAN MM AND PASTAN I. (1987). Expression of a multidrug-resistance gene in human tumors and tissues. *Proc. Natl Acad. Sci. USA*, **84**, 265–269.

FUJII H, TANIGAWA N, MURAOKA R, SHIMOMATSUYA T AND TANAKA T. (1993). Detection of P-glycoprotein in solid tumors by flow cytometry. *Anticancer Res.*, **13**, 2171–2176.

GILL DR, HYDE SC, HIGGINS CF, VALVERDE MA, MINTENING GM AND SEPULVEDA FV. (1992). Separation of drug transport and chloride channel functions of the human multidrug resistance P-glycoprotein. *Cell*, **71**, 23–32.

GOLDSTEIN LJ, GALSKI H, FOJO A, WILLINGHAM M, LAI S-L, GAZDAR A, PIRKER R, GREEN A, CRIST W, BRODEUR GM, LIEBER M, COSSMAN J, GOTTESMAN MM AND PASTAN I. (1989). Expression of a multidrug resistance gene in human cancers. *J. Natl Cancer Inst.*, **81**, 116–124.

GROS P, NERIAH YB, CROOP JM AND HOUSMAN DE. (1986). Isolation and expression of a complementary DNA that confers multidrug resistance. *Nature*, **323**, 728–731.

HAMADA H, HAGIWARA K-I, NAKAJIMA T AND TSURUO T. (1987). Phosphorylation of the $M_r$ 170,000 to 180,000 glycoprotein specific to multidrug-resistant cells: effects of verapamil, trifluoperazine, and phorbol esters. *Cancer Res.*, **47**, 2860–2865.

HIDDEMANN W, SCHUMANN J, ANDREEFF M, BARLOGIE B, HERMAN CJ, LEIF RC, MAYALL BH, MURPHY RF AND SANDBERG AA. (1984). Convention on nomenclature for DNA cytometry. *Cytometry*, **5**, 445–446.

HULTMAN T, STÅHL S, HORNES E AND UHLEN M. (1989). Direct solid phase sequencing of genomic and plasmid DNA using magnetic beads as solid support. *Nucleic Acids Res.*, **17**, 4937–4946.

JASS JR, ATKIN WS, CUZICK J, BUSSEY HJR, MORSON BC, NORTHOVER JMA AND TODD IP. (1986). The grading of rectal cancer: Historical perspectives and a multivariate analysis of 447 cases. *Histopathology*, **10**, 437–459.

JUDSON IR. (1992). Understanding anticancer drug resistance: opportunities for modulation and impact on new drug design. *Eur. J. Cancer*, **28**, 285–289.

KRAMER R, WEBER TK, MORSE B, ARCECI R, STANIUNAS R, STEELE Jr G AND SUMMERHAYES IC. (1993). Constitutive expression of multidrug resistance in human colorectal tumours and cell lines. *Br. J. Cancer*, **67**, 959–968.

LAEMMLI UK. (1970). Cleavage of structural proteins during the assembly of the head of bacteriophage T4. *Nature*, **227**, 680–685.

MOORGHEN M, CAIRNS J, FORRESTER LM, HAYES JD, HALL A, CATTAN AR, WOLF CR AND HARRIS AL. (1991). Enhanced expression of glutathione S-transferases in colorectal carcinoma compared to non-neoplastic mucosa. *Carcinogenesis*, **12**, 13–17.

NOONAN KE, BECK C, HOLZMAYER TA, CHIN JE, WUNDER JS, ANDRULIS IL, GAZDAR AF, WILLMAN CL, GRIFFITH B, VON HOFF DD AND RONINSON IB. (1990). Quantitative analysis of MDR1 (multidrug resistance) gene expression in human tumors by polymerase chain reaction. *Proc. Natl Acad. Sci. USA*, **87**, 7160–7164.

PETERS WHM, BOON CEW, ROELOFS HMJ, WOBBES T, NAGENGAST FM AND KREMERS PG. (1992). Expression of drug-metabolizing enzymes and P-170 glycoprotein in colorectal carcinoma and normal mucosa. *Gastroenterology*, **103**, 448–455.

PIRKER R, WALLNER J, GSUR A, GOTZL M, ZOCHBAUER S, SCHEITHAUER W AND DEPISCH D. (1993). MDR1 gene expression in primary colorectal carcinomas. *Br. J. Cancer*, **68**, 691–694.

PREUDHOMME C, LEPELLEY P, VACHEE A, SOENEN V, QUESNEI B, COSSON A AND FENAUX P. (1993). Relationship between p53 gene mutations and multidrug resistance ($mdr_1$) gene expression in myelodysplastic syndromes. *Leukemia*, **7**, 1888–1890.

REDMOND SMS, JONCOURT F, BUSER K, ZIEMICKI A, ALTERNATT HJ, FEY M, MARGISON G AND CERNY T. (1991). Assessment of P-170 glycoprotein, glutathione-based detoxifying enzymes and 06-alkyl-guanine-DNA alkyltransferase as potential indicators of constitutive drug resistance in human colorectal tumors. *Cancer Res.*, **51**, 2092–2097.

SINICROPE FA, HART J, BRASITUS TA, MICHELASSI F, LEE JJ AND SAFA AR. (1994). P-glycoprotein expression in human colon carcinoma and its relationship to DNA ploidy, local invasion and disease relapse. *Anti-Cancer Drugs*, **5** (Suppl. 1), 57.

SMITH-SORENSEN B, GEBHARDT MC, KLOEN P, MCINTYRE J, AGUILAR F, CERUTTI P, & BORRESEN A-L. (1993). Screening for TP53 mutations in osteosarcomas using constant denaturant gel electrophoresis (CDGE). *Hum. Mutat.*, **2**, 274–285.

UEDA K, CARDARELLI C, GOTTESMAN MM AND PASTAN I. (1987). Expression of a full length cDNA for human 'MDR 1' gene confers resistance to colchicine, doxorubicin and vinblastine. *Proc. Natl Acad. Sci. USA*, **84**, 3004–3008.

WAXMAN DJ. (1990). Glutathione S-transferases: role in alkylating agent resistance and possible target for modulation chemotherapy – a review. *Cancer Res.*, **50**, 6449–6454.

ZHAO B, DRACH D, HU G, SQUIRES J, DRACH J, DEISSEROTH A AND ANDREEFF M. (1992). Absence of regulation of MDR1 by p53 in normal hematopoiesis and acute myelogenous leukemia (abstract). *Blood*, **80** (Suppl.), 797.


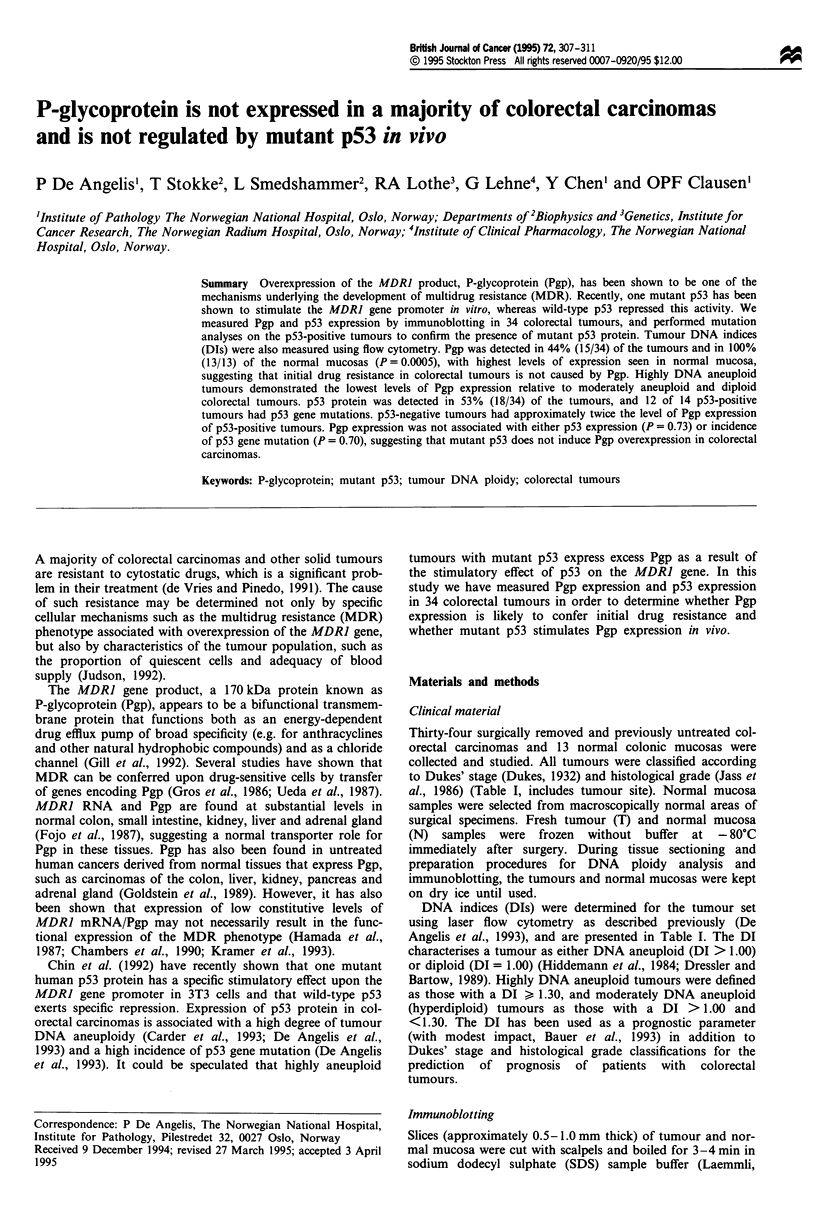

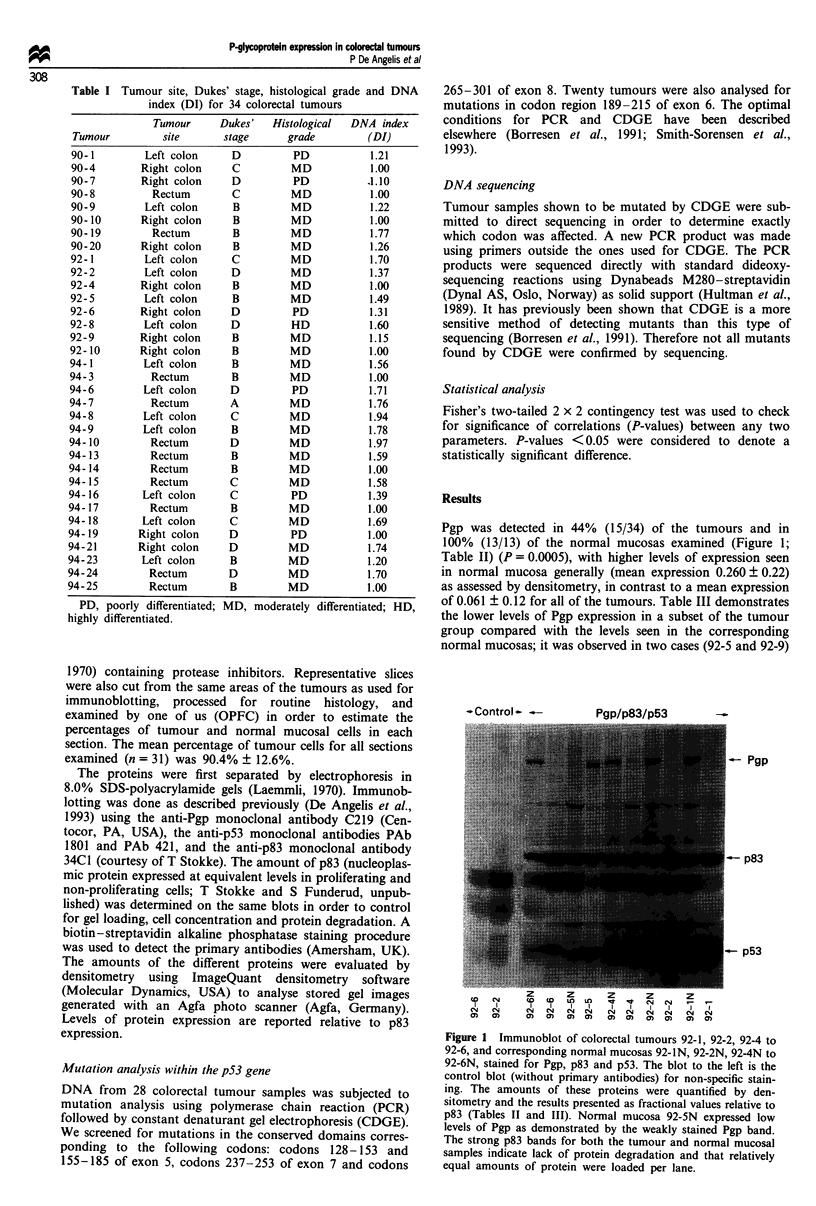

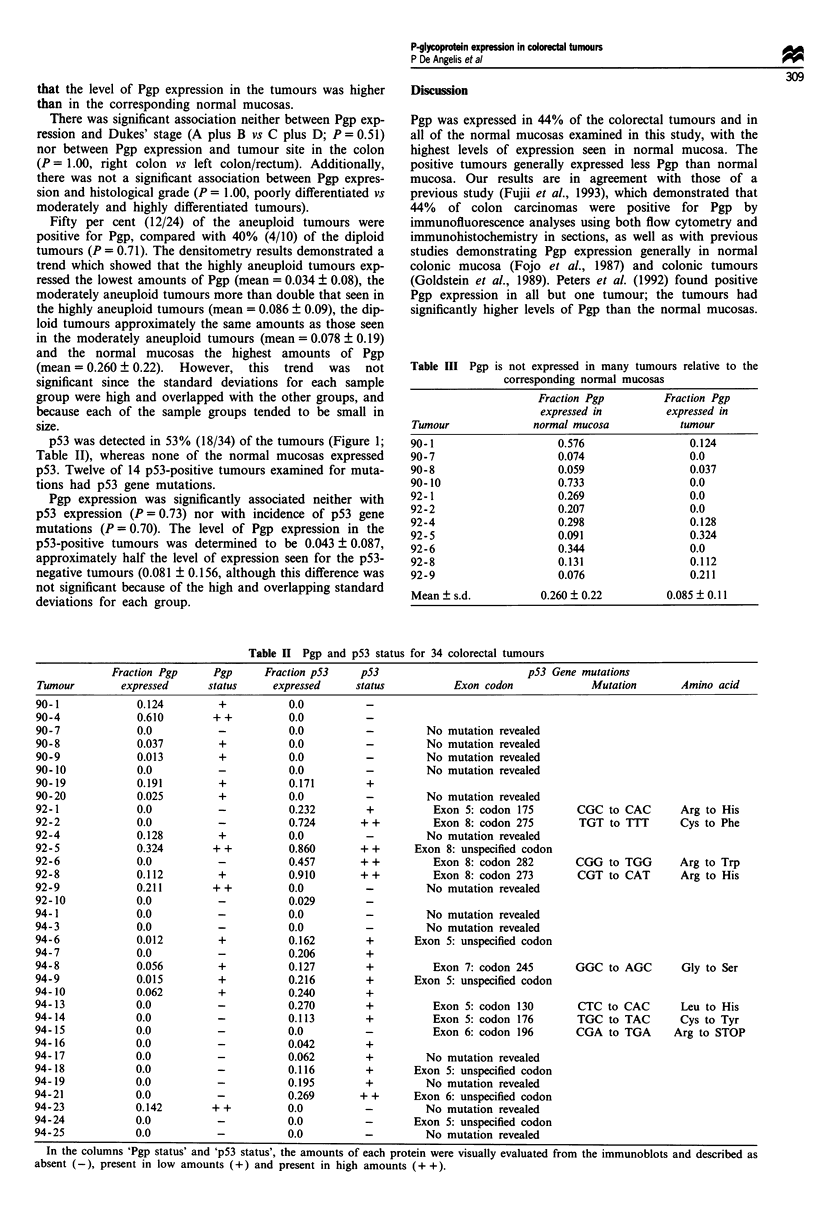

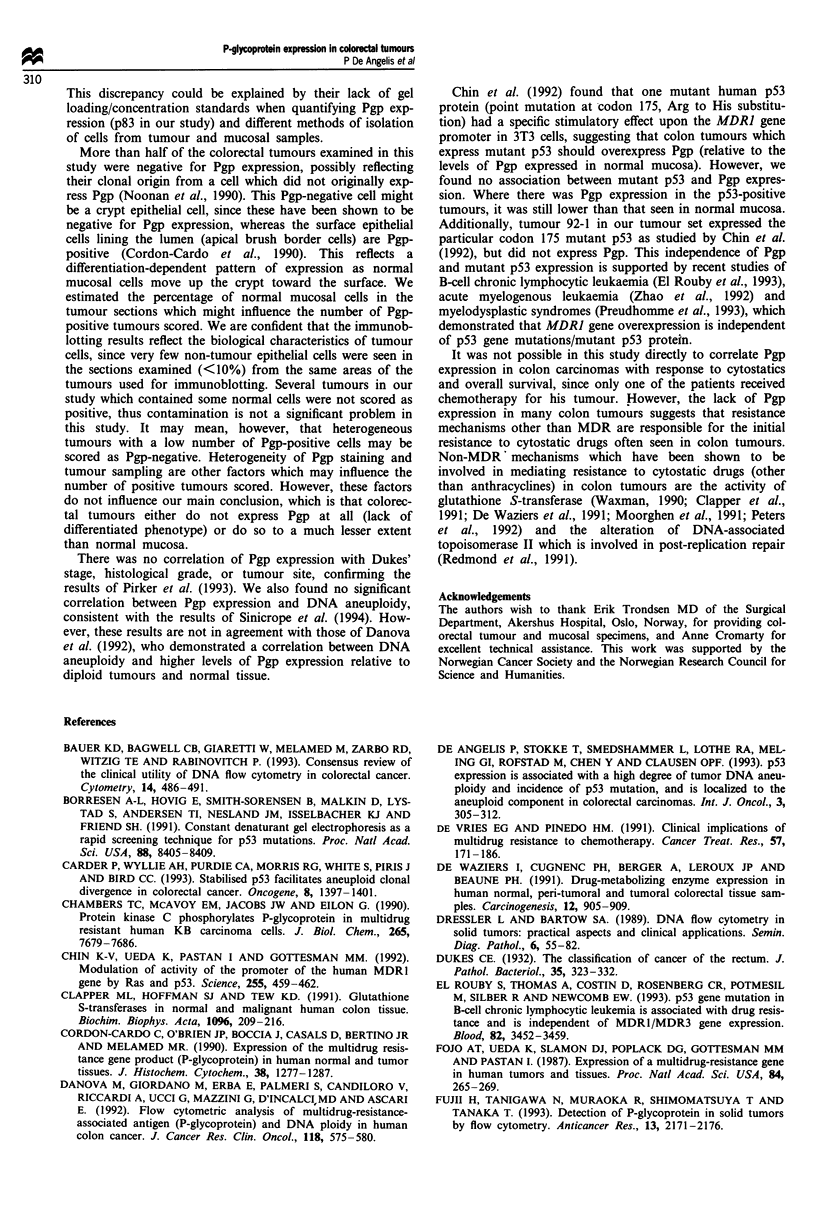

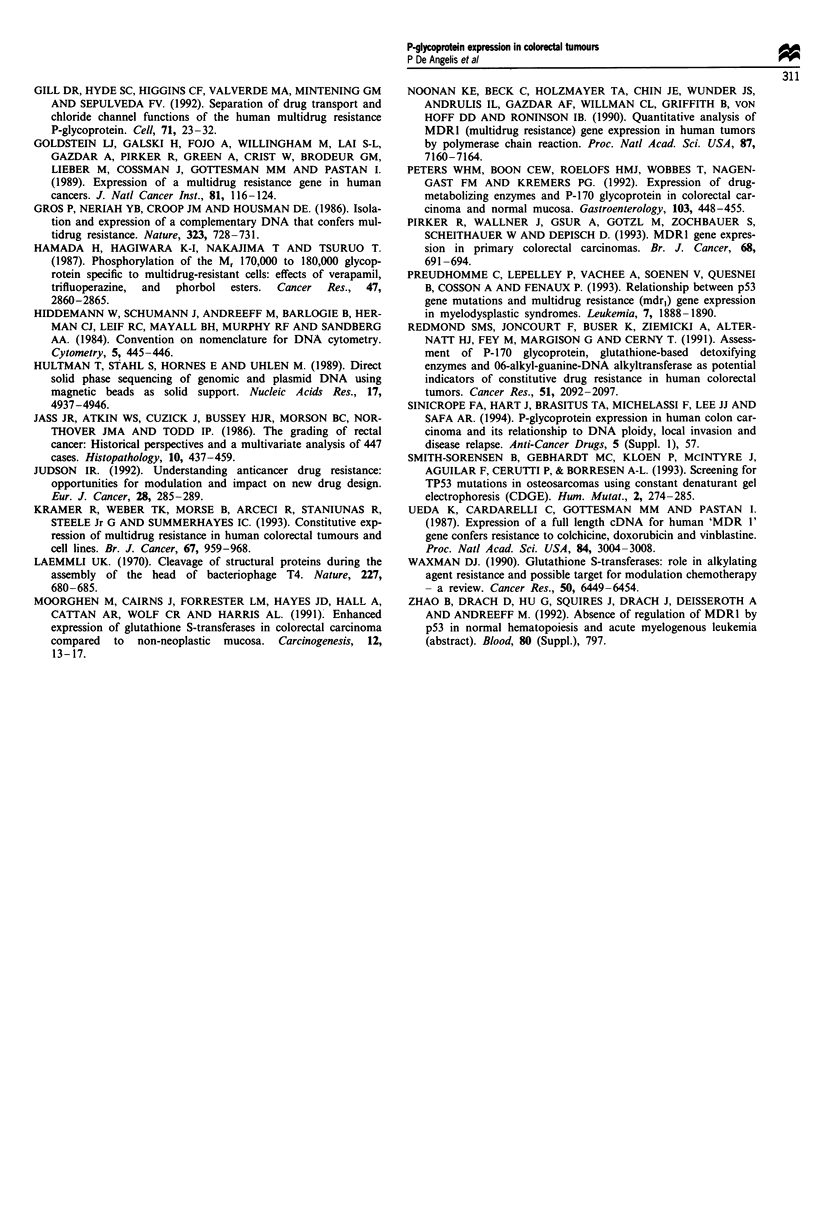


## References

[OCR_00630] Bauer K. D., Bagwell C. B., Giaretti W., Melamed M., Zarbo R. J., Witzig T. E., Rabinovitch P. S. (1993). Consensus review of the clinical utility of DNA flow cytometry in colorectal cancer.. Cytometry.

[OCR_00636] Børresen A. L., Hovig E., Smith-Sørensen B., Malkin D., Lystad S., Andersen T. I., Nesland J. M., Isselbacher K. J., Friend S. H. (1991). Constant denaturant gel electrophoresis as a rapid screening technique for p53 mutations.. Proc Natl Acad Sci U S A.

[OCR_00641] Carder P., Wyllie A. H., Purdie C. A., Morris R. G., White S., Piris J., Bird C. C. (1993). Stabilised p53 facilitates aneuploid clonal divergence in colorectal cancer.. Oncogene.

[OCR_00646] Chambers T. C., McAvoy E. M., Jacobs J. W., Eilon G. (1990). Protein kinase C phosphorylates P-glycoprotein in multidrug resistant human KB carcinoma cells.. J Biol Chem.

[OCR_00652] Chin K. V., Ueda K., Pastan I., Gottesman M. M. (1992). Modulation of activity of the promoter of the human MDR1 gene by Ras and p53.. Science.

[OCR_00659] Clapper M. L., Hoffman S. J., Tew K. D. (1991). Glutathione S-transferases in normal and malignant human colon tissue.. Biochim Biophys Acta.

[OCR_00665] Cordon-Cardo C., O'Brien J. P., Boccia J., Casals D., Bertino J. R., Melamed M. R. (1990). Expression of the multidrug resistance gene product (P-glycoprotein) in human normal and tumor tissues.. J Histochem Cytochem.

[OCR_00671] Danova M., Giordano M., Erba E., Palmeri S., Candiloro V., Riccardi A., Ucci G., Mazzini G., D'Incalci M., Ascari E. (1992). Flow cytometric analysis of multidrug-resistance-associated antigen (P-glycoprotein) and DNA ploidy in human colon cancer.. J Cancer Res Clin Oncol.

[OCR_00694] Dressler L. G., Bartow S. A. (1989). DNA flow cytometry in solid tumors: practical aspects and clinical applications.. Semin Diagn Pathol.

[OCR_00713] Fojo A. T., Ueda K., Slamon D. J., Poplack D. G., Gottesman M. M., Pastan I. (1987). Expression of a multidrug-resistance gene in human tumors and tissues.. Proc Natl Acad Sci U S A.

[OCR_00721] Fujii H., Tanigawa N., Muraoka R., Shimomatsuya T., Tanaka T. (1993). Detection of P-glycoprotein in solid tumors by flow cytometry.. Anticancer Res.

[OCR_00729] Gill D. R., Hyde S. C., Higgins C. F., Valverde M. A., Mintenig G. M., Sepúlveda F. V. (1992). Separation of drug transport and chloride channel functions of the human multidrug resistance P-glycoprotein.. Cell.

[OCR_00736] Goldstein L. J., Galski H., Fojo A., Willingham M., Lai S. L., Gazdar A., Pirker R., Green A., Crist W., Brodeur G. M. (1989). Expression of a multidrug resistance gene in human cancers.. J Natl Cancer Inst.

[OCR_00742] Gros P., Ben Neriah Y. B., Croop J. M., Housman D. E. (1986). Isolation and expression of a complementary DNA that confers multidrug resistance.. Nature.

[OCR_00747] Hamada H., Hagiwara K., Nakajima T., Tsuruo T. (1987). Phosphorylation of the Mr 170,000 to 180,000 glycoprotein specific to multidrug-resistant tumor cells: effects of verapamil, trifluoperazine, and phorbol esters.. Cancer Res.

[OCR_00760] Hultman T., Ståhl S., Hornes E., Uhlén M. (1989). Direct solid phase sequencing of genomic and plasmid DNA using magnetic beads as solid support.. Nucleic Acids Res.

[OCR_00767] Jass J. R., Atkin W. S., Cuzick J., Bussey H. J., Morson B. C., Northover J. M., Todd I. P. (1986). The grading of rectal cancer: historical perspectives and a multivariate analysis of 447 cases.. Histopathology.

[OCR_00770] Judson I. R. (1992). Understanding anticancer drug resistance: opportunities for modulation and impact on new drug design.. Eur J Cancer.

[OCR_00777] Kramer R., Weber T. K., Morse B., Arceci R., Staniunas R., Steele G., Summerhayes I. C. (1993). Constitutive expression of multidrug resistance in human colorectal tumours and cell lines.. Br J Cancer.

[OCR_00783] Laemmli U. K. (1970). Cleavage of structural proteins during the assembly of the head of bacteriophage T4.. Nature.

[OCR_00789] Moorghen M., Cairns J., Forrester L. M., Hayes J. D., Hall A., Cattan A. R., Wolf C. R., Harris A. L. (1991). Enhanced expression of glutathione S-transferases in colorectal carcinoma compared to non-neoplastic mucosa.. Carcinogenesis.

[OCR_00793] Noonan K. E., Beck C., Holzmayer T. A., Chin J. E., Wunder J. S., Andrulis I. L., Gazdar A. F., Willman C. L., Griffith B., Von Hoff D. D. (1990). Quantitative analysis of MDR1 (multidrug resistance) gene expression in human tumors by polymerase chain reaction.. Proc Natl Acad Sci U S A.

[OCR_00803] Peters W. H., Boon C. E., Roelofs H. M., Wobbes T., Nagengast F. M., Kremers P. G. (1992). Expression of drug-metabolizing enzymes and P-170 glycoprotein in colorectal carcinoma and normal mucosa.. Gastroenterology.

[OCR_00810] Pirker R., Wallner J., Gsur A., Götzl M., Zöchbauer S., Scheithauer W., Depisch D. (1993). MDR1 gene expression in primary colorectal carcinomas.. Br J Cancer.

[OCR_00813] Preudhomme C., Lepelley P., Vachee A., Soenen V., Quesnel B., Cosson A., Fenaux P. (1993). Relationship between p53 gene mutations and multidrug resistance (mdr1) gene expression in myelodysplastic syndromes.. Leukemia.

[OCR_00819] Redmond S. M., Joncourt F., Buser K., Ziemiecki A., Altermatt H. J., Fey M., Margison G., Cerny T. (1991). Assessment of P-glycoprotein, glutathione-based detoxifying enzymes and O6-alkylguanine-DNA alkyltransferase as potential indicators of constitutive drug resistance in human colorectal tumors.. Cancer Res.

[OCR_00836] Smith-Sørensen B., Gebhardt M. C., Kloen P., McIntyre J., Aguilar F., Cerutti P., Børresen A. L. (1993). Screening for TP53 mutations in osteosarcomas using constant denaturant gel electrophoresis (CDGE).. Hum Mutat.

[OCR_00841] Ueda K., Cardarelli C., Gottesman M. M., Pastan I. (1987). Expression of a full-length cDNA for the human "MDR1" gene confers resistance to colchicine, doxorubicin, and vinblastine.. Proc Natl Acad Sci U S A.

[OCR_00845] Waxman D. J. (1990). Glutathione S-transferases: role in alkylating agent resistance and possible target for modulation chemotherapy--a review.. Cancer Res.

[OCR_00683] de Vries E. G., Pinedo H. M. (1991). Clinical implications of multidrug resistance to chemotherapy.. Cancer Treat Res.

[OCR_00691] de Waziers I., Cugnenc P. H., Berger A., Leroux J. P., Beaune P. H. (1991). Drug-metabolizing enzyme expression in human normal, peritumoral and tumoral colorectal tissue samples.. Carcinogenesis.

[OCR_00705] el Rouby S., Thomas A., Costin D., Rosenberg C. R., Potmesil M., Silber R., Newcomb E. W. (1993). p53 gene mutation in B-cell chronic lymphocytic leukemia is associated with drug resistance and is independent of MDR1/MDR3 gene expression.. Blood.

